# A Metadynamics-Based Protocol for the Determination of GPCR-Ligand Binding Modes

**DOI:** 10.3390/ijms20081970

**Published:** 2019-04-22

**Authors:** Christian A. Söldner, Anselm H. C. Horn, Heinrich Sticht

**Affiliations:** Bioinformatik, Institut für Biochemie, Emil-Fischer-Centrum, Friedrich-Alexander-Universität Erlangen-Nürnberg (FAU), Fahrstraße 17, 91054 Erlangen, Germany; christian.soeldner@fau.de (C.A.S.); anselm.horn@fau.de (A.H.C.H.)

**Keywords:** receptor-ligand interactions, G protein-coupled receptors (GPCRs), molecular dynamics simulations, metadynamics simulations, ligand binding modes, clustering

## Abstract

G protein-coupled receptors (GPCRs) are a main drug target and therefore a hot topic in pharmaceutical research. One important prerequisite to understand how a certain ligand affects a GPCR is precise knowledge about its binding mode and the specific underlying interactions. If no crystal structure of the respective complex is available, computational methods can be used to deduce the binding site. One of them are metadynamics simulations which have the advantage of an enhanced sampling compared to conventional molecular dynamics simulations. However, the enhanced sampling of higher-energy states hampers identification of the preferred binding mode. Here, we present a novel protocol based on clustering of multiple walker metadynamics simulations which allows identifying the preferential binding mode from such conformational ensembles. We tested this strategy for three different model systems namely the histamine H_1_ receptor in combination with its physiological ligand histamine, as well as the $\beta_{2}$ adrenoceptor with its agonist adrenaline and its antagonist alprenolol. For all three systems, the proposed protocol was able to reproduce the correct binding mode known from the literature suggesting that the approach can more generally be applied to the prediction of GPCR ligand binding in future.

## 1. Introduction

G protein-coupled receptors (GPCRs) are a family of membrane proteins with seven transmembrane helices. There exist more than 800 different members in humans [1,2] most of which are regulated by extracellular ligands. Binding of such a ligand results in structural changes thereby modulating the interaction between the GPCR and its intracellular binding partners like G proteins or β-arrestin [3]. These can in turn regulate a broad range of downstream effectors [2] that influence cellular metabolism [4]. Since GPCRs are involved in the regulation of clinically relevant processes such as for example neurotransmission [5], nociception [6], cardiac function [7], or allergic reactions [8], they are a hot topic in pharmaceutical and medical research. In fact, more than one-third of all FDA-approved drugs target a G protein-coupled receptor [9,10].

To date, there exist more than 250 GPCR crystal structures (including over 50 unique structures) in complex with various ligands providing valuable insights into the structural properties of this protein family. However, due to the large number of receptors and the even larger number of synthetic drug-like ligands, a comprehensive experimental determination of all ligand-GPCR complex structures appears not feasible. For that reason, in silico methods such as docking [11,12] or molecular dynamics (MD) simulations [13,14,15] are of outmost importance to predict the binding site and preferred binding mode of ligands.

Docking is a very popular method because it has the advantage of being very fast, which allows screening multiple ligands in a short time. However, the method has also drawbacks like the limited ability to treat protein flexibility [2,12,16]. This may in particular become problematic if the docked ligands differ significantly from the ligand that was used for the determination of the reference GPCR crystal structure [17]. In addition, most docking methods lack an explicit representation of the solvent and thus fail to detect water-mediated protein-ligand interactions [2].

Molecular dynamics (MD) simulations are capable of considering solvent molecules and protein flexibility; however, they are computationally very expensive. Simulation of ligand binding requires that conventional MD simulations cover at least a microsecond timescale—and even then, ligand binding remains a rare event. For instance, in a previous study where we examined histamine binding to the H_1_ receptor using conventional MD, we performed three simulation runs with a cumulative length of 7 μs, but observed only one single binding event [14]. Dror et al. conducted a similar study, albeit in a much bigger dimension: They performed a set of 82 individual simulations that led to 21 binding events [13], i.e., a success rate of roughly 25%.

For that reason, enhanced sampling methods like metadynamics or adaptive biasing force calculations are more and more applied as an alternative strategy for this objective [18,19,20,21].The concept of metadynamics is to overcome barriers in the free energy landscape by defining one or multiple so-called collective variables (CVs) along which accelerated sampling is desired [22,23,24]. In regular intervals, positive Gaussian potentials, which are centered at the current location of the system within the CV space, are added to the energy landscape. This history-dependent bias potential discourages the system from sampling configurations that have already been visited before and allows for an efficient exploration of the free energy surface as a function of the CVs [22]. For metadynamics simulations of ligand binding, it has proven useful to introduce an additional funnel-shaped bias potential which restrains the ligand within a certain distance of the binding path [25]. That way, sampling of irrelevant regions outside the receptor can be avoided which leads to a further increase of efficiency.

To date, metadynamics simulations of GPCRs have been successfully used to simulate binding of ligands in a couple of cases including for example opioid receptors [21,26], vasopressin [20], chemokine [27], or cannabinoid receptors [19]. Due to the high accuracy in sampling energy landscapes [24,28], the method has so far mainly been used to calculate binding free energies and less emphasis was put on the detailed analysis of the underlying ligand binding mode. In this context, it has to be noted that the determination of the lowest-energy binding mode (i.e., the most stable ligand binding pose) is not straightforward. While the history-dependent bias potential that is used in metadynamics [23,24] leads to sampling of low-energy states, the ligand does not remain fixed at these positions. Instead, it samples repeatedly also higher-energy states making it difficult to determine the preferred binding mode by visual inspection.

Here, we present a clustering-based strategy, which facilitates the detection of the most stable ligand binding mode from GPCR metadynamics simulations. This approach relies on the refinement of representative structures from the most populated clusters, observed in the metadynamics simulation, by conventional MD simulations. The protocol was tested for the binding of histamine and adrenaline to the physiological receptors, histamine H_1_ receptor (H_1_R) and $\beta_{2}$ adrenoceptor ($\beta_{2}$AR), respectively. In addition, binding of the antagonist alprenolol to the $\beta_{2}$AR was investigated to assess whether the strategy also works for synthetic ligands. To render the test cases more realistic, the following considerations were made:

(i) Inclusion of ligands that share limited structural similarity with the co-crystallized ligand (histamine binding was simulated starting from H_1_R structure crystallized with doxepin).
(ii) Use of starting structures that were crystallized in a different activation state (binding of the agonist histamine was simulated starting from the structure of the inactive H_1_R; binding of the antagonist alprenolol was simulated starting from the structure of active $\beta_{2}$AR).
(iii) Use of μs-MD simulations in the absence of the ligand for equilibration of the free GPCR structure (performed for all systems).

The respective setups are summarized in Table 1, which states both the crystal structure used as a starting structure of the simulations and the reference structure, which was used for the identification of the correct binding mode. For all three ligands, the presented clustering protocol allowed detecting the preferential binding mode and performed significantly better than a conventional docking protocol. Thus, we conclude that the metadynamics-based clustering protocol represents a versatile approach that can also be applied to other GPCR-ligand complexes in future.

## 2. Results and Discussion

### 2.1. Overview of the Computational Strategy

**Figure 1:**
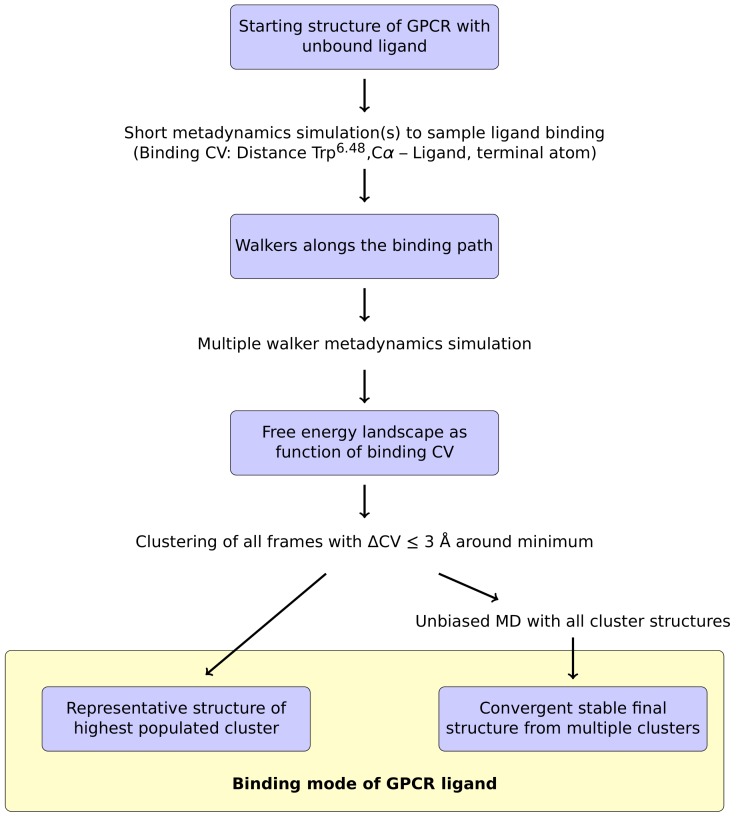
Overview of the proposed metadynamics-based protocol for the determination of ligand modes to GPCRs. See text for a detailed description.

For the metadynamics simulation itself, we adapted the protocol of Saleh et al. [28], which relies on a common collective variable (CV) to study GPCR-ligand interactions. In this approach, the *z* component of the distance between the ammonium nitrogen atom (or a functionally equivalent atom) of the ligand and the Cα atom of the conserved Trp^6.48^ at the bottom of the binding pocket is selected as CV [28]. To apply this protocol to the prediction of ligand binding modes, one conceptual change had to be made: Saleh et al. mainly investigated the energy landscape of the binding process, which allowed starting the simulations from the ligand-bound GPCR structure [28]. However, an ab initio prediction of ligand binding modes requires to start from an unbound ligand. For that purpose, the ligand was placed at a random position within the solvent above the receptor and one or multiple short initial metadynamics runs were performed to sample the binding path of the ligand to the GPCR. A brief summary of this approach is shown in Figure 1.

From the trajectories obtained in these initial simulations, sample conformations are extracted along the whole binding path, which are then used as initial structures for the multiple walker simulation (Figure 1). Based on this multiple walker simulation, the free energy profile along the binding CV is calculated. The resulting curve should have a global minimum at the energetically most favorable CV value. The next step in the suggested protocol is to extract all frames from the whole set of multiple walker trajectories which are located within a certain interval around the CV value of the free energy minimum (e.g., ΔCV ≤ 3 Å) in order to perform a subsequent clustering analysis. After an RMSD fit of the protein backbone, the extracted frames are clustered according to the ligand conformation. Based on these clusters, it was tested whether the ligand binding mode can already be reliably detected using a representative structure from the highest populated cluster (Figure 1). Alternatively, unbiased MD simulations were used for representative structures from each cluster to allow for conformational adjustment within the ligand binding site. In the following sections, we present the results that were obtained for the testing systems using the computational protocol described above.

### 2.2. Metadynamics Simulations and Free Energy Profile

As shown in Figure 1, the approach starts from an unbound ligand in the solvent and short initial metadynamics simulations are used to extract sample conformations along the binding path of the ligand. Such a simulation is provided exemplarily for the binding of histamine to the H_1_ receptor as Supplementary Materials, Video S1. For this system, 25 ns of simulation time were sufficient to observe multiple reversible entries from an initial position within the solvent into the orthosteric binding site. For adrenaline and alprenolol, the additional introduction of an upper wall for the CV was required to avoid the escape of the ligands into the bulk solvent thereby facilitating ligand entrance into the orthosteric binding site resulting in a more even distribution of the walkers along the CV (see Methods for details).

**Figure 2:**
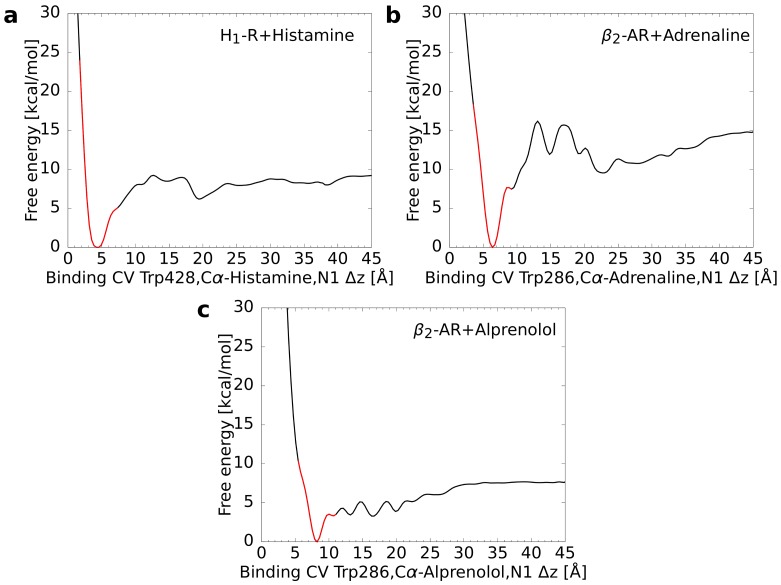
Free energy as a function of the binding CV calculated from the multiple walker metadynamics simulations. In each graph, the global minimum was set to zero. The part of the curve, for which the CV difference ΔCV to the minimum is less or equal to 3 Å, is colored in red. (**a**) Histamine in complex with the H_1_ receptor, (**b**) adrenaline, and (**c**) alprenolol both in complex with the $\beta_{2}$ adrenoceptor.

For each of the three test systems, 32 starting structures were extracted, which were equidistantly distributed with respect to the binding CV. Based on these structures multiple walker simulations were performed that reached a cumulative simulation time of approximately 1500 ns (32 × 48 ns) per system. For these simulations, the free energy profile was calculated as a function of the binding CV. This approach led always to a pronounced global minimum representing the energetically most favorable CV value (Figure 2). It was checked subsequently whether the location of these minima was in accordance with the values calculated for the reference structures (i.e., complex crystal structures in case of the $\beta_{2}$AR ligands [30,31] and an experimentally validated model in case of histamine H_1_R [14]). For every system, the difference between the free energy minimum of the metadynamics simulation and the reference was smaller than 0.5 Å suggesting a rather good agreement (Table 2).

### 2.3. Clustering of the Frames around the Free Energy Minimum

**Figure 3:**
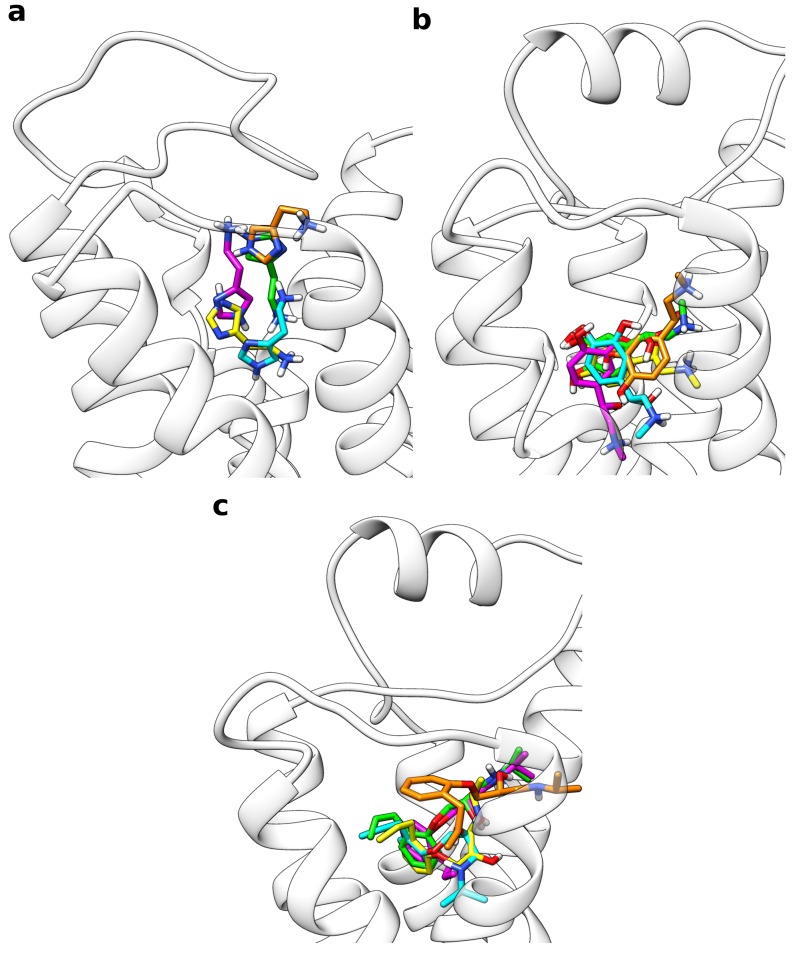
Overlay of the representative structures from all five ligand clusters calculated from the multiple walker simulations. With decreasing percentage of the frames, for which they are representative, the structures are colored in yellow (most populated), green, cyan, magenta, and orange (least populated). (**a**) Histamine in complex with the H_1_ receptor, (**b**) adrenaline, and (**c**) alprenolol both in complex with the $\beta_{2}$ adrenoceptor.

Following the presented protocol, all frames from the multiple walker trajectories were extracted, which were closer than 3 Å to the free energy minimum according to their CV value. Based on this approach, depending on the system, 15.6 to 22.0% of all frames were taken into account as can be seen from Table 2. This set of conformations was then subjected to clustering based on the ligand orientation with respect to the receptor backbone. A final target of five structure clusters per receptor was defined. For all systems investigated, this strategy resulted in a set of rather different ligand orientations within the binding site (Figure 3).

For example, in the case of histamine, the ammonium group was oriented into completely reversed directions in different clusters. A common feature observed for all sample systems was, however, that there occurred always one predominantly populated cluster, which was representative for a high proportion of the extracted frames (Table 3). For the system consisting of histamine and the H_1_ receptor, about 92% of all snapshots were assigned to cluster 1. Single-digit percentages were calculated for clusters 2 and 3 whereas the last two clusters contained less than 1% of the frames. Table 3 also lists the RMSD of the respective cluster structures compared to the reference binding mode. With 1.35 Å, the histamine RMSD is the lowest for cluster 1 and much higher (3.5 to 8.3 Å) for the other clusters. For adrenaline and alprenolol at the $\beta_{2}$ receptor, the percentage of frames belonging to the first cluster was a bit lower with approximately 70% in both cases given that about one fifth of the snapshots were assigned to cluster 2. For both systems, the fraction of cluster 3 was between 4 and 9% while the last two clusters were almost negligible. Again, as reported for histamine, the RMSD of cluster 1 to the reference binding mode was very low with 0.42 Å in case of adrenaline and 0.99 Å in case of alprenolol. Cluster 2 still had a moderate RMSD of approximately 2 Å for both ligands whereas the deviation of all other clusters was higher. For all cluster representatives in all test systems the RMSD values of the orthosteric pocket are rather low (Table 3) indicating that the metadynamics simulations do not cause large changes of the pocket geometry.

**Figure 4:**
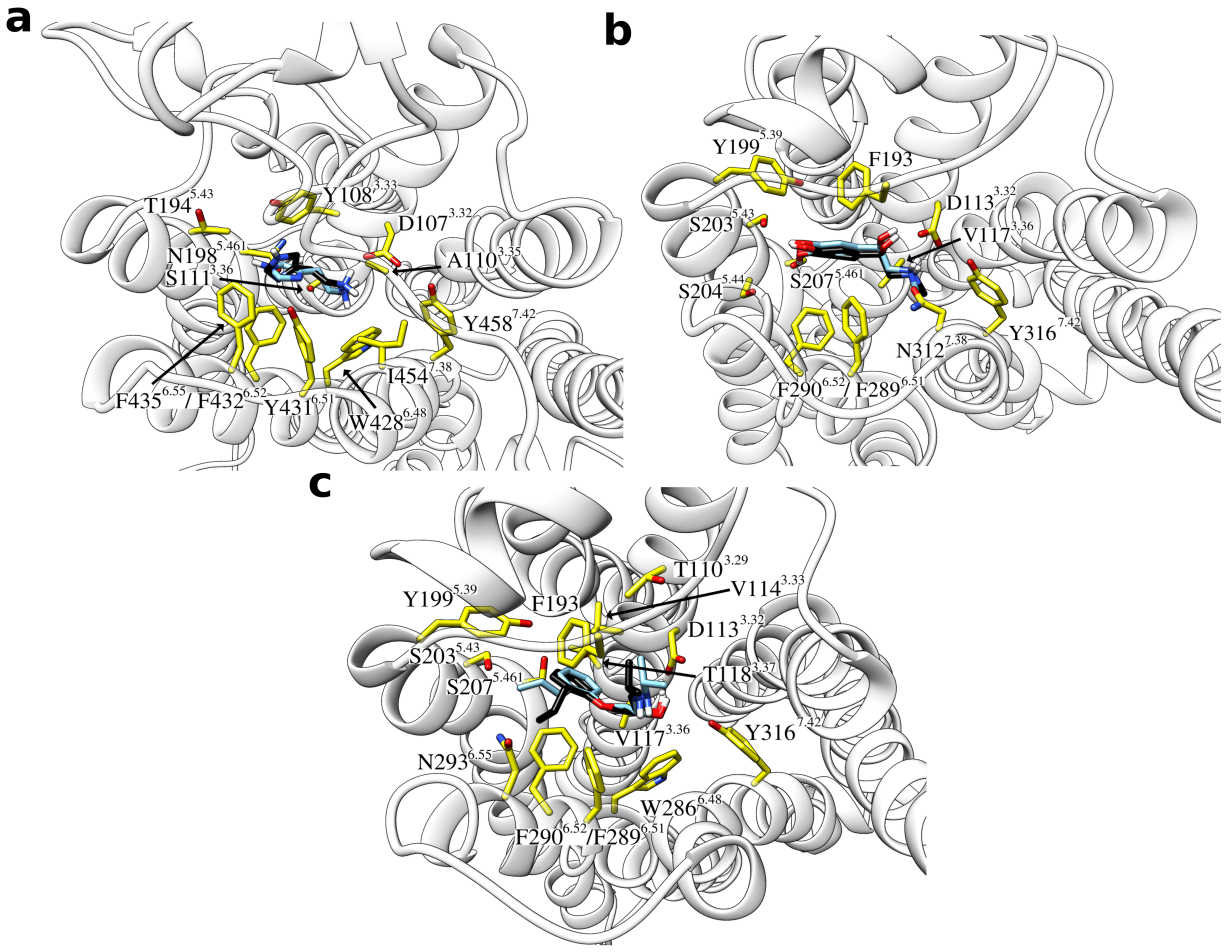
Overlay of the most populated ligand cluster structures and the reference binding mode. The ligands are shown as sticks with the carbon atoms of the reference ligand colored in black and those of the cluster structure in light blue. Interacting residues of the respective GPCR are labeled and shown as yellow sticks. (**a**) Histamine in complex with the H_1_ receptor, (**b**) adrenaline, and (**c**) alprenolol both in complex with the $\beta_{2}$ adrenoceptor.

Taken together, there was thus one predominantly populated cluster structure in all sample systems, which was characterized by a high similarity to the reference binding mode. An overlay of these most frequent clusters with the reference binding modes is provided in Figure 4. In all these cases, the general ligand orientation within the binding site is correct and contacts are formed with the same GPCR residues.

### 2.4. Unbiased MD Simulations of the Cluster Representatives

**Figure 5:**
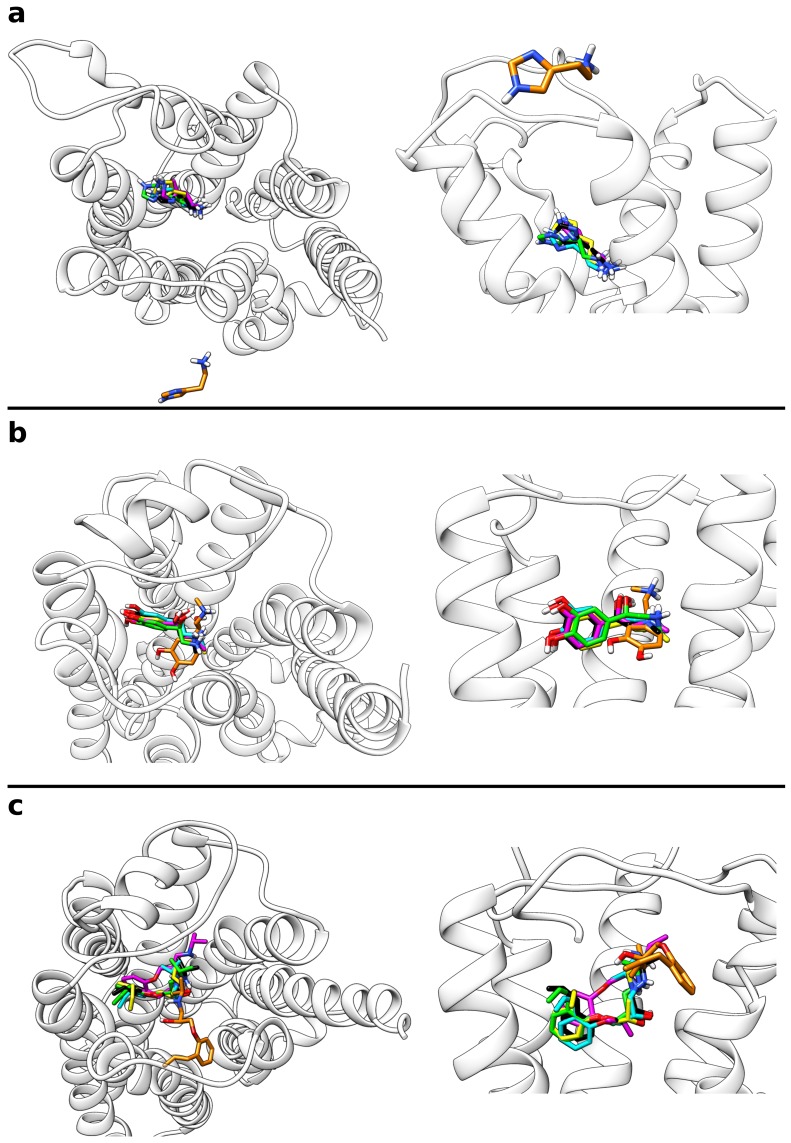
Overlay of all five final cluster structures from the unbiased MD simulations with the reference binding mode (left: top-view, right: zoomed-in lateral view). The ligands are shown as sticks with the carbon atoms of the reference ligand colored in black and those of the cluster structures colored in yellow (most populated), green, cyan, magenta and orange (least populated) according to the color scheme described in Figure 3. (**a**) Histamine in complex with the H_1_ receptor, (**b**) adrenaline, and (**c**) alprenolol both in complex with the $\beta_{2}$ adrenoceptor.

**Figure 6:**
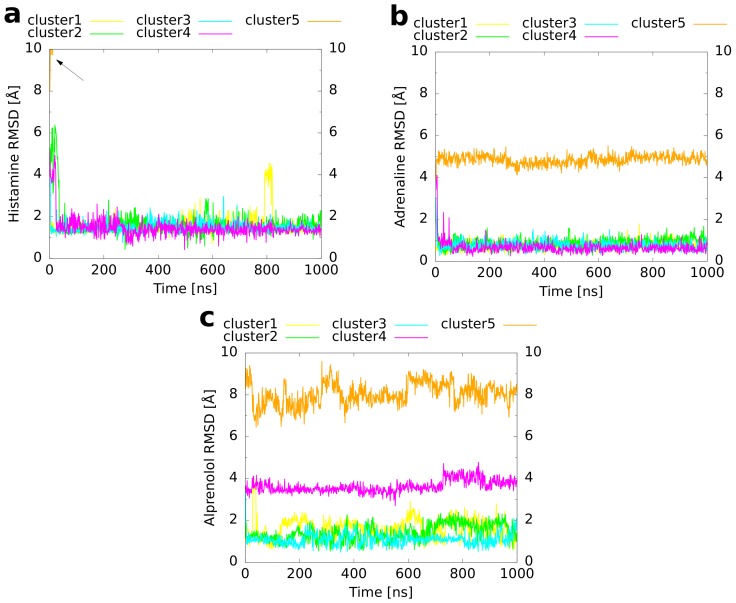
RMSD of the ligand heavy atoms compared to the reference binding mode. The RMSD is plotted as a function of simulation time for the unbiased MD simulations of the cluster representatives. A preceding fit to the protein backbone, but not to the ligand coordinates was performed. (**a**) Histamine in complex with the H_1_ receptor, (**b**) adrenaline, and (**c**) alprenolol both in complex with the $\beta_{2}$ adrenoceptor. The arrow in panel (**a**) marks the rapid increase of the histamine RMSD of cluster 5 due to the dissociation of the ligand from the receptor.

**Figure 7:**
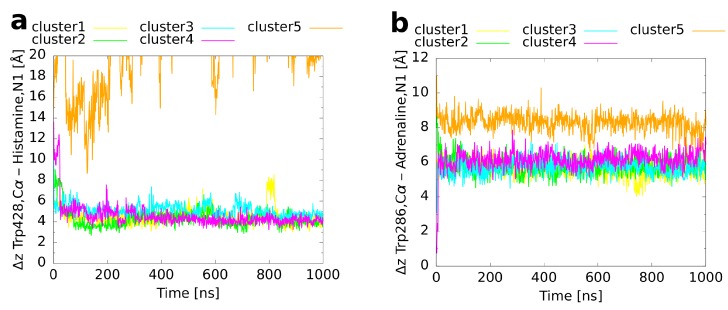

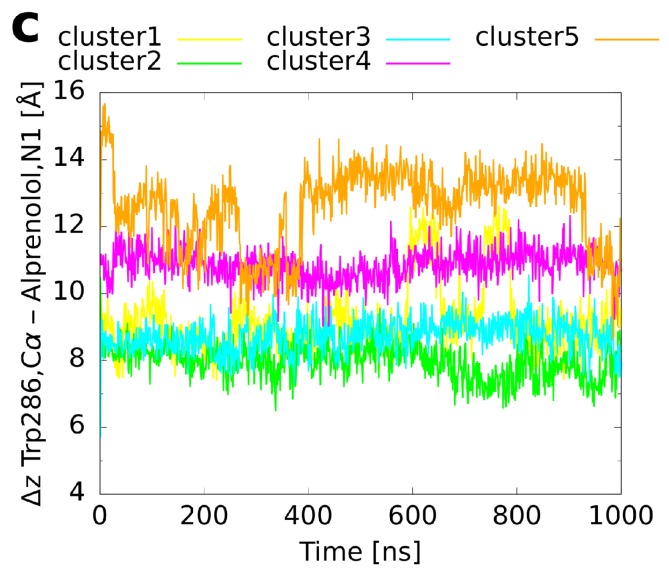
Binding CV (*z* distance between the Trp^6.48^ Cα atom and the ligand’s amine/amino nitrogen atom) as a function of simulation time in the unbiased MD simulations of the cluster representatives. (**a**) Histamine in complex with the H_1_ receptor, (**b**) adrenaline, and (**c**) alprenolol both in complex with the $\beta_{2}$ adrenoceptor.

In order to refine the binding modes of the cluster representatives and to investigate whether they remain conformationally stable, an unbiased molecular dynamics simulation of 1 μs simulation time was performed for every cluster. An overlay of the final structures from these MD runs (Figure 5) with the reference binding mode shows for histamine (Figure 5a) that four of the simulations led to a very similar orientation of the ligand within the binding pocket. For cluster 5, in contrast, a dissociation of histamine was observed. The convergence of clusters 1 to 4 is also visible from a plot of the histamine RMSD with respect to the reference binding mode as a function of simulation time (Figure 6a). While cluster 1 showed a nearly constant RMSD of 1–2 Å during the whole simulation, the RMSDs of clusters 3–4 decreased rapidly to the same level during the first 50 ns. Moreover, the convergence of the first four clusters becomes apparent from a plot of the binding CV (Figure 7a) over the simulation time, which approached quickly the distance of the reference binding mode (4.6 Å) and remained afterwards constant except for some variation around the mean. The unbinding of cluster 5 happened directly at the beginning and was followed by a rapid increase of the RMSD (indicated by an arrow in Figure 6a). These differences most likely result from the fact that cluster 5 had already initially the highest RMSD of all clusters (Table 3) reflecting that histamine was located farther outside than in the other cluster representatives (Figure 3a and Figure 7a).

The results obtained for adrenaline are very similar. As for histamine, four of the five cluster structures converged to a binding mode which was in good agreement with the reference (Figure 5b). As the only exception, cluster 5, which had a particularly inclined conformation in the binding pocket with the amine nitrogen atom pointing upwards and the hydroxy groups at the aromatic ring pointing downwards (Figure 3b), did not rotate within the simulated time span. Again, the RMSD (Figure 6b) of cluster 1 remained rather constant during the whole simulation time whereas the values for clusters 2–4 decreased in the first 20 ns to the same level of ≈1 Å. For cluster 5, the RMSD always fluctuated around 5 Å without major changes during the simulation. As expected, in clusters 1–4, the value of the binding CV (Figure 7b) approached that of the reference (6.6 Å) whereas it was a bit higher (≈8 Å) for cluster 5 due to the comparatively steep orientation of adrenaline within the binding pocket.

For alprenolol, which was the biggest ligand investigated, only clusters 1–3 converged to the reference binding mode (Figure 5c). Again, as shown for histamine and adrenaline, this happened immediately at the beginning of the respective runs (Figure 6c). In contrast, clusters 4–5 did not reach the same conformation. Cluster 4 remained always a bit farther outside than the reference (Figure 7c, reference 8.2 Å) with the ligand oriented too steep in the binding pocket. Although cluster 5 was the farthest from the orthosteric binding site at the beginning (Figure 3c and Figure 7c), it descended over time showing throughout a pronounced flexibility. However, it twisted eventually and the aromatic ring was trapped in the interface between transmembrane helices 5 and 6 so that it could not converge to the binding mode of the reference.

Taken together, it can be concluded for all systems investigated that the highly populated clusters always converged to the binding conformation of the reference already in the first 50 ns of the respective MD trajectories. Only for cluster structures that were representative for less than 2% of the extracted frames, exceptions were found. These exceptions included once a complete dissociation, twice the adoption of an alternative binding mode, which remained conformationally stable during the investigated time span, and once a persisting conformational flexibility within the binding pocket.

### 2.5. Assessment of the Suggested Strategy

Above, we have presented a metadynamics-based strategy for the determination of GPCR-ligand binding modes. Our approach was able to reproduce the known binding modes for all three considered sample systems. As outlined in the introduction, the general idea to use metadynamics for this purpose is not entirely new. In the past, this method has already been successfully applied for a couple of systems such as opioid [26] or cannabinoid receptors [19]. However, the main focus of the previous studies were mostly energetic analyses of the binding mode [18,19,26] whereas generally less emphasis was given to the deduction of a representative conformation for the resulting receptor-ligand complex. Our suggestion to use a clustering strategy for this objective, is one step to extend the application of metadynamics farther towards the investigation of structural properties. In the following, we would like to set the proposed protocol into context with alternative methods and discuss the strength and weaknesses of the approach.

Compared to conventional MD simulations which aim to sample spontaneous ligand binding starting from a ligand position within the extracellular solvent, the concept to investigate the binding path with an enhanced sampling method should increase efficiency. Metadynamics is an algorithm that was developed to accelerate sampling of rare events, which would otherwise be unlikely to happen on the accessible time scales [23]. Ligand binding to GPCRs is one example for such a rare event, which occurs with on-rates in the range of $10^{6}$–$10^{10}$ M${}^{-1}\cdot$min${}^{-1}$ [32]. Consequently, binding events are only rarely sampled or may not be detected at all on a microsecond timescale, which is typically used to study such processes by conventional MD [13,14].

In addition, as described by Limongelli et al., the funnel used in the metadynamics simulations will decrease irrelevant sampling within the solvent thereby further enhancing the chance of ligand binding [25]. The present study suggests that the shape of the funnel originally proposed by Saleh et al. [28] can also be applied when starting from an unbound ligand in the solvent. However, we noted that a tighter upper wall on the CV may be required as an additional modification to enhance binding probability during the initial metadynamics simulation from which the walkers along the binding path are extracted. We expect that this type of modification will in particular be required for larger ligands in future, which may exhibit reduced binding propensities because binding frequently requires conformational rearrangements of the GPCR along the binding pathway and in the binding pocket.

Although metadynamics may be more efficient than conventional MD, it nevertheless remains a very expensive technique. One multiple walker simulation with 32 walkers and a cumulative simulation time of 1500 ns required about 150,000 core hours on the Intel Xeon E5-2630v4 cluster (2.2 GHz) that was used to perform most of our simulations. The costs are up to three times higher if ternary complexes (GPCR, ligand, and G protein) are studied because they require a bigger membrane patch and water box [33]. Following our original protocol, additional CPU time may be necessary to perform the subsequent unbiased MD runs of the cluster representatives (in our case about 60,000 core hours per system and μs). This step may eventually be omitted, since the present study suggests that the highest populated cluster structures directly obtained from the metadynamics simulation are already very close to the correct binding mode and may be refined by very short (<100 ns) MD simulations. However, future studies for additional ligands will be required to prove that this observation is generally valid. Moreover, it should always be checked if the obtained binding mode fits to data from the literature such as for example mutagenesis studies from the GPCRdb [34].

The significantly higher computational costs of the metadynamics approach compared to conventional docking protocols prompted us to compare the performance of both methods with respect to their accuracy. Docking started from the same crystal structures as the metadynamics simulations (Table 1) and AutoDock Vina [35] with standard settings was used for docking (see Supplementary Materials File S1 for details).

The best ligand RMSDs obtained for adrenaline, alprenolol, and histamine docking are 2.2 Å, 8.4 Å, and 5.1 Å, respectively (File S1). Thus, for all three systems, the metadynamics-based approach produced better ligand poses (Table 3) than a standard molecular docking procedure (File S1). The differences are most prominent for those systems, in which the ligand (histamine, alprenolol) was docked to a receptor structure corresponding to a different activation state. The importance of the GPCR activation state for the outcome of docking studies has also been reported previously by Beuming et al. [17]. In contrast, the present protocol appears rather robust with respect to the activation state of the GPCR starting structure.

The observation that docking of histamine and alprenolol produced only solutions, which differ significantly from the correct binding mode, also renders their use as starting points for a refinement by conventional MD problematic. Such an approach has been previously suggested by Bartuzi et al. [36] and would allow saving computer time by replacing the metadynamics step by conventional docking. However, our work also demonstrates that the success of the conventional MD for refinement depends on the quality of the initial ligand poses generated. This is exemplified by alprenolol, which converges to the correct binding mode for clusters 1–3, which exhibit a low RMSD after the metadynamics step, but not for clusters 4 + 5 (Figure 6). Thus, the metadynamics step may only be replaced by conventional docking, if docking is capable of producing solutions that are sufficiently close to the correct binding mode to allow successful refinement by conventional MD. In our study, this approach might therefore work for adrenaline, but has a high risk to fail for histamine and alprenolol because no good docking solutions were obtained for these ligands (File S1).

Although the present metadynamics-based protocol might be still too compute-intensive for large-scale drug screening studies without further optimization, its good performance renders it a promising approach for projects where only a limited number of ligands is investigated. In addition to its accuracy, it offers further advantages compared to docking simulations: For example, information about the free energy landscape and the binding pathway is obtained from the multiple walker simulation [28]. This data might in addition to the mere binding mode of the ligand also be useful for drug development because the design of ligands which enter the receptor more easily could lead to an improved drug efficacy [13].

Based on the considerations above, we feel that the metadynamics-based protocol developed in the present study offers a valuable supplement in the toolbox of theoretical methods for the study of GPCR-ligand interactions.

## 3. Methods

### 3.1. Systems Investigated and Preparation of Starting Structures

An overview of the systems investigated including the atoms used for the definition of the binding CV is provided in Table 4.

The protocol used to set up histamine and the H_1_ receptor is described in our previous publication [14]. The geometry for the ligands adrenaline and alprenolol was taken from the respective PDB entries 4LDO [30] and 3NYA [31]. Hydrogen atoms were added using Avogadro 1.1 [37]. With antechamber from AmberTools 17 [38], *gaff* [39] atom types were assigned. For adrenaline, partial charges were derived by means of a RESP/ESP fit with the RESP/ESP Charge Derive (R.E.D.) Server [40] using Firefly 7.1 [41,42] and the base set RESP-C2 (HF/6-31G*//HF/6-31G*). In case of alprenolol, AM1-BCC charges [43] were assigned with antechamber because previous studies have shown that AM1-BCC yields charge sets of comparable quality to HF/6-31G${}^{*}$ ESP-derived charges in a fraction of the time [44]. Amber Prep files for histamine, adrenaline, and alprenolol are provided in the Supplementary Materials as Files S2, S3, and S4, respectively. By means of the AmberTool tleap, coordinate and topology files for the ligands were created and afterwards converted to Gromacs file formats using amb2gmx.pl [45].

All simulations for the $\beta_{2}$ adrenoceptor were set up based on the PDB entry 4LDO [30]. Everything except for the GPCR was removed (adrenaline, the engineered nanobody, T4 lysozyme, …). The unresolved gap between Gln231 and Lys263 was filled with a glycine-serine spacer (sequence GSGS) using ModLoop [46,47]. The incomplete termini were capped with an N-terminal acetyl and a C-terminal N-methyl group, which were added using Sybyl7.3 [48]. With tleap, the system was prepared for MD: Missing hydrogen atoms were created, disulfide bonds were set as specified in the PDB file, and the *ff99SB* force field [49] was used. In order to avoid steric tension, an initial energy minimization of the protein was performed before membrane embedding using a TIP3P water box (capped octahedron, distance from solute to borders ≤ 8 Å, Cl${}^{-}$ ions for neutralization). This minimization (500 steps *steepest descent* + 4500 steps *conjugate gradient*) was done with *sander* from Amber17. After removing water and ions, new Amber topology and coordinate files were created for the minimized protein, which were afterwards again converted to Gromacs file formats. The protein was then overlaid with a preequilibrated dioleoylphosphatidylcholine (DOPC) membrane [50] (gaff force field, solvated with SPC water [51]) according to the entry 4LDO from the OPM database [52]. The overlay was performed using an in-house Perl script that minimized the squared *z* components of the distances between the DOPC C1 atoms and the pseudoatoms, by which the position of the membrane borders is represented in the OPM entry. After combining coordinates and topologies for protein and membrane, membrane embedding was performed with gmx membed [53]. Then, Cl${}^{-}$ ions were added for electrical neutralization using gmx genion.

Next, a three-step energy minimization was conducted with gmx mdrun. First, all atoms except for water molecules and ions were restrained using harmonic potentials with a force constant of 1000 kJ·mol${}^{-1}\cdot$nm${}^{-2}$ in *x*, *y*, and *z* direction. For the second step, only the Cα atoms of the GPCR were kept restrained with the same force constant. Then, in the third phase, the complete system was minimized. All three steps comprised an initial part using the *steepest descent* algorithm, which was followed by a second part with *conjugate gradient* minimization. The runs were terminated when machine precision was reached, which was usually the case after a few thousands of steps.

To equilibrate the embedded receptor in the DOPC bilayer, 300 short consecutive MD simulations with 100 ps length were conducted. Position restraints were imposed on the GPCR backbone using the same force constant as for the minimization. Before the next simulation of this series was started, all water molecules that had entered the space between protein and membrane, were removed with an in-house Perl script. Finally, an unrestrained 2 μs simulation was performed to dispose of structural artifacts which might be a result of the co-crystallization with the T4 lysozyme and the engineered nanobody.

### 3.2. Molecular Dynamics Simulations

For all unbiased MD simulations, Gromacs 2016.5 [54] was used. By means of a Berendsen thermostat [55], the temperature was held at 310 K. The system was divided in the three coupling groups solvent+ions, protein+ligand, and the DOPC membrane. All simulations were performed at constant pressure using surface-tension coupling at a reference *z* pressure of 1 bar and a reference surface tension of 1.1 nm·bar. In *x*, *y*, and *z* direction, periodic boundary conditions were applied. Since bonds involving hydrogen atoms were constrained using the LINCS algorithm [56], a time step of 2 fs was chosen. All subsequent trajectory analyses were conducted with cpptraj (AmberTools 17) [57]. For the clustering analysis, we created a pseudotrajectory and performed an RMSD fit on the protein backbone. Then, the hierarchical agglomerative (bottom-up) clustering approach based on an unfitted RMSD of the ligand heavy atoms was chosen. The structures of the cluster representatives that were obtained for histamine, adrenaline, and alprenolol are provided within the Supplementary Materials as File S5, S6, and S7, respectively. gnuplot [58] was used to create plots and structures were visualized with UCSF Chimera [59].

### 3.3. Metadynamics Simulations

A detailed description of the metadynamics protocol is provided in [14]. All metadynamics simulations were conducted using Gromacs 2016.3 [54] with the plumed 2.3.1 [60] plugin. Well-tempered metadynamics simulations were performed according to an established strategy reported by Saleh et al. [28]. Since the membrane patches with the GPCRs were arranged parallel to the x/y plane of the coordinate system, the *z* component of the distances between the Cα atoms of the conserved residues Trp^6.48^ and the amino/amine nitrogen atoms of the investigated aminergic ligands could be used as CV. For the multiple walker simulations of the H_1_ receptor, lower and upper walls for the CV were defined at $z_{\mathrm{low}}=0.3$nm and $z_{\mathrm{up}}=4.9$ nm, respectively. In case of the $\beta_{2}$ adrenoceptor, they were located at $z_{\mathrm{low}}=0.4$ nm and $z_{\mathrm{up}}=4.5$ nm. For the walls, harmonic potentials with a force constant κ=1500 kJ·mol${}^{-1}\cdot$nm${}^{-2}$ were set:

(1) $${\mathrm{Bias}}_{\mathrm{low}}=\left\{\begin{array}{cc} \kappa \cdot {\left(z-z_{\mathrm{low}}\right)}^{2} & \mathrm{for}\;z\leq z_{\mathrm{low}}\\ 0 & \mathrm{for}\;z>z_{\mathrm{low}} \end{array}\right.\;{\mathrm{Bias}}_{\mathrm{up}}=\left\{\begin{array}{cc} 0 & \mathrm{for}\;z<z_{\mathrm{up}}\\ \kappa \cdot {\left(z-z_{\mathrm{up}}\right)}^{2} & \mathrm{for}\;z\geq z_{\mathrm{up}} \end{array}\right.$$

To reduce irrelevant sampling within the bulk solvent, a bell-shaped funnel was applied in x/y direction. The center of this funnel was located at the Trp^6.48^ Cα atom. Depending on the CV value *z*, the radius R(z) of the funnel is given as

(2) $$R\left(z\right)=\frac{d}{1+\exp\left(m\cdot \left(z-w\right)\right)}+r_{1}$$

with d=1.6 nm, $r_{1}=0.8$ nm, m=10 nm${}^{-1}$, and w=2.8 nm. If $r\left(x,y\right)=\sqrt{x^{2}+y^{2}}$ is the xy projection of the distance between the ligand’s amino/amine nitrogen atom and the Trp^6.48^ Cα atom, the funnel-restraint has the form

(3) $$\mathrm{Funnel}\;\mathrm{bias}\left(x,y,z\right)=h\cdot \left(1-\frac{1}{1+\exp\left(\lambda \cdot \left(r\left(x,y\right)-R\left(z\right)\right)\right)}\right)$$

where h=100 kJ·mol${}^{-1}$ and λ=500.

For metadynamics, the same general parameters (temperature, pressure coupling, time step etc.) were used as for the unbiased MD simulations. The initial metadynamics runs to sample the binding pathway of the ligands were performed with bias factor 50, an initial bias height of 7 kJ·mol${}^{-1}$ and a gaussian width of 0.1. In case of insufficient sampling of the binding pathway (as observed for adrenaline and alprenolol), the ligands were placed directly at the entry of the binding pocket and an upper wall for the CV was introduced at that position. This setup avoids the escape of the ligands into the bulk solvent and enhances the population inside the binding pocket. To obtain walkers along the entire binding path, conformations outside the binding pocket were supplemented from a separate simulation run. For the multiple walker simulations, the bias factor was reduced to 20 and the initial bias height was set to 5 kJ·mol${}^{-1}$. To calculate the free energy landscape from the HILLS files, the plumed shell command was used.

## Figures and Tables

**Table 1:** Overview of the systems investigated including the starting structures and the reference binding modes used for the validation of the suggested protocol. Note that all starting structures were equilibrated for more than 2 μs without a ligand after membrane insertion (for details see “Methods”). H_1_R denotes the histamine H_1_ receptor, $\beta_{2}$AR the $\beta_{2}$ adrenoceptor.

System	Starting Crystal Structure	Reference Binding Mode
H_1_R + histamine	PDB 3RZE (crystallized with doxepin) [29]	unbiased MD study [14]
$\beta_{2}$AR + adrenaline	PDB 4LDO (crystallized with adrenaline) [30]	crystal structure PDB 4LDO [30]
$\beta_{2}$AR + alprenolol	PDB 4LDO (crystallized with adrenaline) [30]	crystal structure PDB 3NYA [31]

**Table 2:** Energy minima of the multiple walker metadynamics simulations. The CV values for the global free energy (FE) minima from the simulations are compared with the CV values measured for the reference structures described in Table 1. Moreover, the table lists the percentages of frames from the multiple walker trajectories, which have a CV value within 3 Å of these minima.

System	CV of Reference	CV of FE Minimum	Frames with ΔCV ≤ 3 Å
H_1_R + histamine	4.3 Å	4.6 Å	15.6%
$\beta_{2}$AR + adrenaline	6.6 Å	6.4 Å	22.0%
$\beta_{2}$AR + alprenolol	8.6 Å	8.2 Å	16.8%

**Table 3:** Clustering of the multiple walker metadynamics trajectories around the free energy minima. The table contains the percentages of those frames within 3 Å around the minima, for which the calculated clusters are representative, and the RMSD values of the cluster structures compared to the reference binding modes. The RMSD values refer to heavy atoms and were calculated both for the ligand and for the residues of the orthosteric binding site, i.e., the residues which are located within 5 Å of the ligand in the reference structure.

System	Cluster Number	Percentage of Frames	RMSD Compared to Reference [Å]	
			Ligand	Receptor Binding Pocket
H_1_R + histamine	1	91.7	1.35	1.78
	2	5.3	5.29	1.81
	3	2.3	3.46	1.75
	4	0.6	5.39	1.92
	5	0.2	8.33	1.77
$\beta_{2}$AR + adrenaline	1	74.0	0.42	0.40
	2	21.0	1.96	0.48
	3	4.6	3.06	0.62
	4	0.4	4.38	0.72
	5	0.1	3.75	0.57
$\beta_{2}$AR + alprenolol	1	69.1	0.99	0.70
	2	20.6	2.43	0.61
	3	8.5	3.40	0.70
	4	1.7	3.43	0.48
	5	0.1	8.20	0.61

**Table 4:** Overview of the systems investigated. The table lists the atoms between which the binding collective variable (CV) was defined as well as the total number (♯) of atoms, water molecules, and DOPC molecules in the system.

System	CV: Δz Distance	♯ Atoms	♯ Water	♯ DOPC
H_1_R + histamine	Trp428^6.48^,Cα–histamine,N1	98,075	20,347	235
$\beta_{2}$AR + adrenaline	Trp286^6.48^,Cα–adrenaline,N1	98,271	20,431	234
$\beta_{2}$AR + alprenolol	Trp286^6.48^,Cα–alprenolol,N1	98,236	20,415	234

## Supplementary Materials

Supplementary Materials can be found at https://www.mdpi.com/1422-0067/20/8/1970/s1. Video S1: Short metadynamics simulation sampling the binding pathway of histamine to the H_1_ receptor. File S1: Comparison of the metadynamics-based protocol with conventional docking. File S2: Amber Prep file for histamine. File S3: Amber Prep file for adrenaline. File S4: Amber Prep file for alprenolol. Files S5-S7: PDB files with the structures of the cluster representatives for histamine (File S5), adrenaline (File S6), and alprenol (File S7) before refinement by MD simulations.
