# Supplementary material for: A Metadynamics-Based Protocol for the Determination of GPCR-Ligand Binding Modes
Source: Int J Mol Sci. 2019 Apr 22;20(8):1970. doi: 10.3390/ijms20081970 (PMC6514967; doi:10.3390/ijms20081970)
Supplement: Supplementary file 1 [file ijms-20-01970-s001.zip › ijms-482180-supplementary-2/file_s1_comparison_with_docking.pdf]

Supplementary Information

Comparison of the metadynamics-based protocol with conventional docking

Christian A. Söldner, Anselm H. C. Horn & Heinrich Sticht

1 Supplementary Results for the Molecular Docking

The metadynamics-based protocol suggested in this publication is computationally rather expensive. Thus, we wanted to check for our testing systems whether similar results can be obtained using a conventional docking approach, which would require much less computational resources. We chose the same GPCR crystal structures for docking that were also used for the metadynamics simulations (main paper, Table 1).

For the β_2AR, we used the PDB structure 4LDO [1] which had been crystallized in complex with adrenaline. Redocking of adrenaline to this structure, a so called bound docking, should therefore be comparatively easy. Indeed, three of the ten best-energy docking solutions had an RMSD of 2–3 Å compared to the reference ligand binding mode (Table S1). However, the representative of the most populated cluster from the metadynamics-based protocol even showed a lower RMSD of only 0.4 Å (main paper, Table 2).

For alprenolol both metadynamics and docking started from the crystal structure of adrenaline-bound β_2AR (PDB: 4LDO). This is the more challenging case of binding to a receptor crystallized with a different ligand and even in a different activation state. For this system, the RMSD values of the lowest-energy docking poses compared to the reference (PDB 3NYA [2]) were between 8.4 and 17.5 Å (Table S1) and thus rather large from the crystallographic binding mode. In contrast, the metadynamics-based protocol led to an RMSD of 1.0 Å for the highest populated cluster representative (main paper, Table 2).

In case of histamine and the H_1 receptor, docking was performed to the PDB structure 3RZE [3], which had been crystallized in complex with doxepin. The structures of histamine and doxepin differ significantly from each other; the tricyclic doxepin is much larger with a molar mass of 279 g/mol compared to 111 g/mol for histamine [4]. The docking poses of histamine showed RMSD values between 5.1 and 6.8 Å (Table S1) compared to the reference [5], which is more than three times higher than the RMSD of 1.4 Å that was calculated for the most populated cluster representative in the metadynamics protocol (main paper, Table 2).

2 Supplementary Methods

Docking was performed with AutoDock Vina 1.1.2 [6]. AutoDock Tools [7] were used for the initial preparation of receptors and ligands, which included the assignment of AutoDock atom types and Gasteiger charges [8]. The search space for the ligand binding site was defined as a rectangular box with the dimensions 20 Å × 20 Å × 20 Å in x, y, and z direction. The center of the box was set using the coordinates of the Trp$^{6.48}$ Cα atom with the z coordinate increased by 5 Å to match approximately the center of the orthosteric pocket. The receptors were kept rigid and the default exhaustiveness of 8 was used. For every system, the 10 docking solutions with the best free energy of binding were considered.

Table S1: Docking results. Heavy atom RMSD of the ligand compared to the reference binding mode and free energy of binding according to AutoDock Vina.

System	Number of pose	RMSD to reference [Å]	Free energy of binding [kcal/mol]
β_2AR+adrenaline	1	2.84	−7.5
	2	5.21	−6.2
	3	11.15	−6.1
	4	9.34	−6.1
	5	11.05	−6.0
	6	11.43	−6.0
	7	10.58	−6.0
	8	2.21	−5.9
	9	10.08	−5.8
	10	2.64	−5.8
β_2AR+alprenolol	1	9.01	−7.3
	2	8.45	−7.0
	3	16.87	−3.9
	4	16.81	−3.9
	5	17.45	−3.9
	6	16.15	−3.9
	7	16.79	−3.8
	8	16.20	−3.8
	9	16.55	−3.8
	10	16.44	−3.8
H_1R+histamine	1	6.70	−4.8
	2	6.80	−4.5
	3	5.71	−4.3
	4	6.38	−4.3
	5	6.24	−4.1
	6	5.82	−4.1
	7	5.95	−4.1
	8	5.10	−4.0
	9	5.73	−3.8
	10	5.27	−3.7

References

[1] A. M. Ring, A. Manglik, A. C. Kruse, M. D. Enos, W. I. Weis, K. C. Garcia, and B. K. Kobilka. "Adrenaline-activated structure of β2-adrenoceptor stabilized by an engineered nanobody". In: *Nature* 502.7472 (Sept. 2013), pp. 575–579. DOI: 10.1038/nature12572.

[2] D. Wacker, G. Fenalti, M. A. Brown, V. Katritch, R. Abagyan, V. Cherezov, and R. C. Stevens. "Conserved Binding Mode of Human β2Adrenergic Receptor Inverse Agonists and Antagonist Revealed by X-ray Crystallography". In: *Journal of the American Chemical Society* 132.33 (Aug. 2010), pp. 11443–11445. DOI: 10.1021/ja105108q.

[3] T. Shimamura, M. Shiroishi, S. Weyand, H. Tsujimoto, G. Winter, V. Katritch, R. Abagyan, V. Cherezov, W. Liu, G. W. Han, T. Kobayashi, R. C. Stevens, and S. Iwata. "Structure of the human histamine H1 receptor complex with doxepin". In: *Nature* 475.7354 (June 2011), pp. 65–70. DOI: 10.1038/nature10236.

[4] G. Pándy-Szekeres, C. Munk, T. M. Tsonkov, S. Mordalski, K. Harpsøe, A. S. Hauser, A. J. Bojarski, and D. E. Gloriam. "GPCRdb in 2018: adding GPCR structure models and ligands". In: *Nucleic Acids Research* 46.D1 (Nov. 2017), pp. D440–D446. DOI: 10.1093/nar/gkx1109.

[5] C. A. Söldner, A. H. C. Horn, and H. Sticht. "Binding of histamine to the H1 receptor—a molecular dynamics study." In: *Journal of molecular modeling* 24 (12 Nov. 2018), p. 346. ISSN: 0948-5023. DOI: 10.1007/s00894-018-3873-7.

[6] O. Trott and A. J. Olson. "AutoDock Vina: Improving the speed and accuracy of docking with a new scoring function, efficient optimization, and multithreading". In: *Journal of Computational Chemistry* (2009), NA–NA. DOI: 10.1002/jcc.21334.

[7] G. M. Morris, R. Huey, W. Lindstrom, M. F. Sanner, R. K. Belew, D. S. Goodsell, and A. J. Olson. "AutoDock4 and AutoDockTools4: Automated docking with selective receptor flexibility". In: *Journal of Computational Chemistry* 30.16 (Dec. 2009), pp. 2785–2791. DOI: 10.1002/jcc.21256.

[8] J. Gasteiger and M. Marsili. "A new model for calculating atomic charges in molecules". In: *Tetrahedron Letters* 19.34 (Jan. 1978), pp. 3181–3184. DOI: 10.1016/s0040-4039(01)94977-9.
